# Ocean Color Indices of Frontal Boundary Movements: A Multi-Sensor View

**DOI:** 10.3390/s26175641

**Published:** 2026-09-04

**Authors:** Jason K. Jolliff, M. David Lewis, Sherwin D. Ladner, T. Adam Lawson

**Affiliations:** U.S. Naval Research Laboratory, Washington, DC 20375, USA; mark.d.lewis54.civ@us.navy.mil (M.D.L.); sherwin.d.ladner.civ@us.navy.mil (S.D.L.); timothy.a.lawson24.civ@us.navy.mil (T.A.L.)

**Keywords:** ocean color, continental shelves, ocean dynamics, coastal ocean, ocean fronts

## Abstract

Strong and optically conspicuous frontal boundaries are common in the river-dominated Louisiana–Texas Shelf (LTS). Variable wind stress forcing along cross-shelf density gradients results in convergences/divergences that may lead to rapid vertical water mass displacements. In cases where near-bottom shelf waters are displaced to the surface, the associated optical anomaly is often distinct in satellite ocean color data. A satellite-observed optical feature consistent with near-bottom-water ventilation is examined over the LTS with multiple satellite-based ocean color radiometers (OLCI, VIIRS), as well as data from the Advanced Baseline Imager (ABI) on the geostationary GOES-R platform. In the aftermath of an atmospheric cold front passage and sustained northerly winds, the combined satellite analysis reveals a rapidly westward moving optical front delineated by a sharp visible-band reflectivity gradient. Ocean model simulations reproduce a qualitatively similar displacement of surface density fields that is consistent with advection by the along-front current with a potentially modulating influence from the diurnal heating. This study serves as an example of the kind of analyses that may be possible from future geostationary ocean color missions.

## 1. Introduction

Ocean studies in the satellite era have greatly benefitted from the synoptic nature of remote sensing data, and the biogeochemical consequences of large-scale divergence in ocean circulation are very clearly discerned by ocean color sensors [1,2]. However, a geostationary orbit for space-borne ocean monitoring sensors is the exception [3] rather than the rule as many Earth-observing satellites have a polar-orbiting configuration [4,5]. The present constellation of polar-orbiting radiometers specifically designed for ocean monitoring may indeed provide multiple datasets for a designated ocean area in a single day, but the consistency required to resolve oceanic processes at very high temporal resolution is not guaranteed.

High-temporal-resolution datasets may be required to improve the study of ocean water mass boundaries, ocean fronts, which are of primary importance for a wide range of physical and biogeochemical ocean processes [6]. Ocean fronts in coastal regions tend to be zones of intense vertical water movements (upwelling/downwelling), and so are vital conduits for the movement of heat, salt, nutrients and other biogeochemically pertinent materials [7,8,9,10]. Due to the often amplified biological activity and transport of materials along fronts, they may be observed with satellite-based radiometers calibrated to detect variation in water-leaving radiance (ocean color) [11].

These frontal boundaries encompass a wide range of time and space scales: from quasi-permanent ocean features [12] to comparatively ephemeral phenomena that persist for less than a day [13,14]. Thus, the ability to monitor and study ocean fronts with remote sensing is biased towards the spatiotemporal resolution of the data collected by spaceborne sensors. At the frequency of 1 or 2 passes per day and a spatial ground resolution of 300 to 750 m, the present ocean color constellation is capably detecting many frontal boundary zones [11,15,16]. However, physical processes along frontal boundaries are often rapid, sometimes lasting only hours and concentrated over fine-scale ocean gradients (<1 km) [17]. These ocean *submesoscale* processes (meters to 10 km and hours to days [18]) may not be reliably resolved with the present ocean-viewing radiometer suite.

A geostationary platform is better suited to study diurnal variance of submesoscale ocean features [14]; however, there is presently no ocean color geostationary-based sensor in the Western Hemisphere. Radiometers do reside on the meteorological Geostationary Operational Environmental Satellites (GOES) platforms. The ABI sensor on the GOES-16 (East) platform has two visible-band channels (470 and 640 nm) that are designed to detect clouds and atmospheric aerosols [19]. The signal-to-noise ratio for these ABI bands is below what is nominally required for ocean color studies [20], which requires isolation of the comparatively faint water-leaving radiance signal (L_w_). Nonetheless, the very high surface water reflectance values in some coastal scenes, due in part to high concentrations of suspended particulates, may be detected by the ABI visible bands [21,22,23,24]. Due to the very high temporal imaging resolution afforded by the geostationary platform (5 min), ABI has the potential to aid in the study of complex, high-frequency coastal ocean processes in ways that may not feasible with standard, polar-orbiting-based sensors.

In this paper, we identify a frontal boundary over the LTS that was detectable by satellite on 21 March 2019. Very high reflectance contrast values were observed where we hypothesize that significant upward vertical water mass movements were occurring on the seaward side of shelf fronts. Very high reflectance values in these coastal scenes also enabled rudimentary front detection by the GOES-16 ABI instrument at 500 m horizontal resolution (ABI band 2) and 1 km resolution (ABI band 1). The combination of detailed spectral information provided by the polar-orbiting ocean color sensors (VIIRS, OLCI) and the high-temporal-resolution geostationary ABI data permit a detailed investigation of the optics and physical dynamics of the shelf fronts. This study provides a demonstration of this technique as well as a preview of what may be possible with the fusion of sensors of increasing spectral (NASA PACE [25]) and temporal (geostationary GLMIR [26]) resolution in present and future satellite ocean color missions.

**Figure 1:**
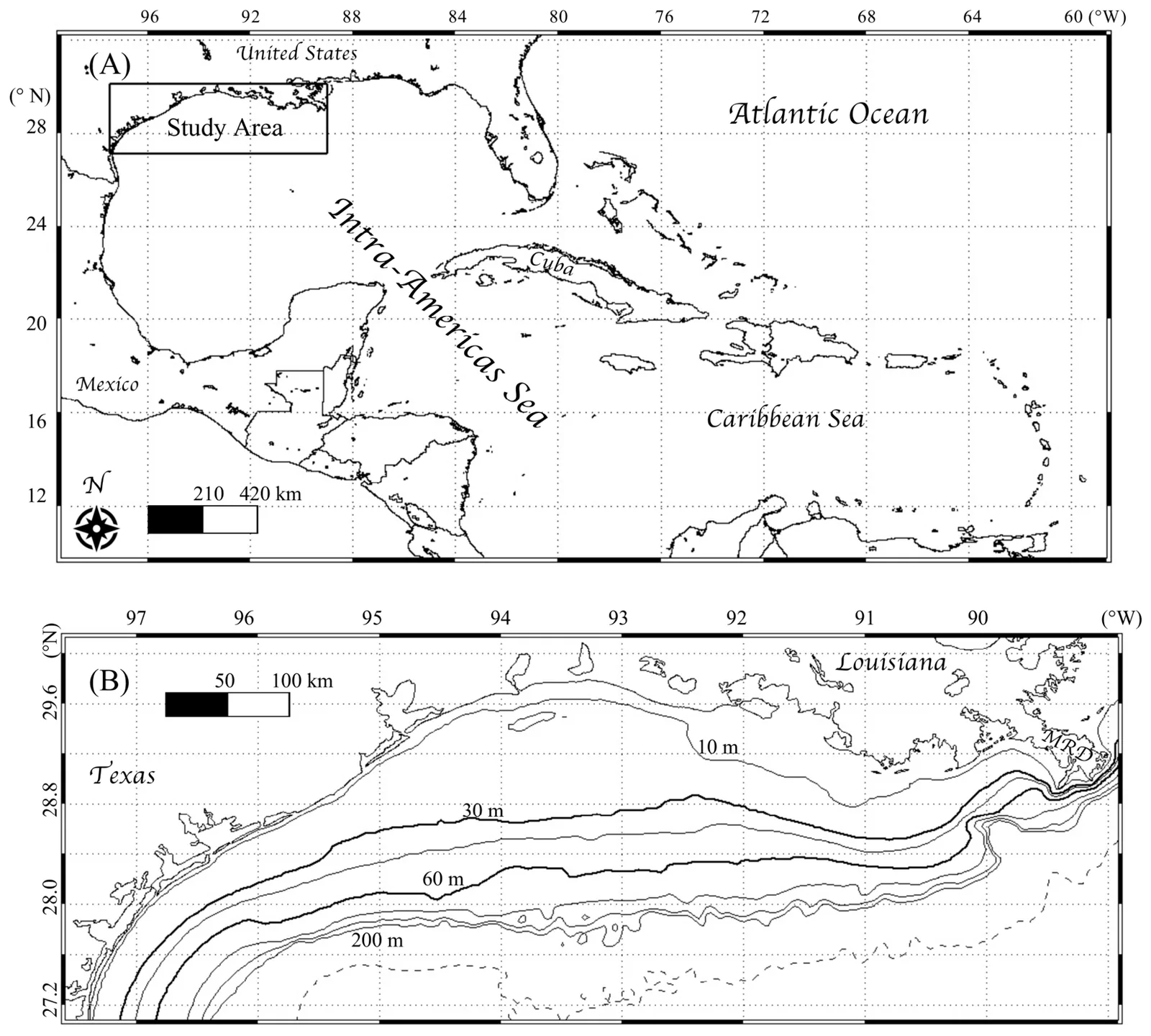
(**A**) Map of Intra-Americas Sea (IAS) with study area indicated in the top left. (**B**) Map of the Louisiana–Texas Shelf (LTS) with bathymetry contours overlaid. Bathymetry is the NRL DBDB2 2 min bathymetry grid.

The study area (Figure 1) encompasses the northern portion of the Intra-Americas Sea (IAS), which is an all-encompassing term for the portion of the eastern Atlantic basin circulation through the Caribbean’s Windward Islands and up into the semi-enclosed basin along the Mexican and United States coasts via the Yucatan Straits. In this paper, the LTS refers specifically to the continental shelf (<200 m depth) west of the Mississippi River Delta and extending west and then south to the Texas–Mexico border (Figure 1). The dominant pelagic circulation feature in the northern IAS is the Loop Current and associated mesoscale eddies that propagate towards the west [27,28,29]. The LTS circulation is dominated by the freshwater outflow from the Mississippi–Atchafalaya river systems [30,31,32,33] and seasonal transitions between spring/summer thermohaline stratification and autumn/winter vertical mixing episodes.

A significant component of the LTS seasonal oceanographic dynamics are frequent autumn/winter episodes of continental cold air outbreak (CAO) events [34,35,36,37,38,39], which are characterized by a ~10 °C drop in air temperatures and strong, sustained northerly winds. Thus, prevailing surface currents over the LTS are often towards the west as a result of both buoyancy forcing (from river discharge) and the westward Ekman transport response to northerly wind forcing [40,41,42]. This analysis is focused on the aftermath of a CAO event that occurs at the winter to spring transition when strong northerly winds and relatively cloud-free skies permitted the retrieval of multiple datasets from satellite-based passive radiometers.

## 2. Materials and Methods

The satellite ocean color datasets were obtained from the Ocean and Land Colour Imager (OLCI) on board the Sentinel-3A/3B satellites and the Visible Infrared Imaging Radiometer Suite (VIIRS) instruments on the Suomi-National Polar-Orbiting Partnership (SNPP) and NOAA-20 satellites. OLCI and VIIRS Level-1 sensor data were processed using the latest version of the Naval Research Laboratory’s (NRL) Automated Optical Processing System (AOPS) [43]. An intermediate processing radiance, [surface reflectance “rhos”; *ρ*_s_], was used to generate the true color scenes. The rhos Level-2 product is the target reflectance remaining after correction for Raleigh scattering, ozone, absorption, and water vapor absorption, as described in NASA ocean-color-processing technical documentation [44].

Standard Level-3 remote sensing product data were rendered via the full atmospheric (aerosol) correction procedure in AOPS. Inherent Optical Properties (IOPs) were rendered via the Quasi-Analytic Algorithm (QAA) [45]. The original version of the algorithm computed the spectral total absorption and backscattering coefficients. An extension of the QAA was used to also retrieve the total scattering coefficient (b, m^−1^), and by addition, the beam attenuation coefficient (*b* + *a* = *c*).

True color fields shown in this paper were constructed following the colorimetric method [46,47]. Surface reflectance from the sensors at each available visible band (OLCI, 10; VIIRS, 5) were interpolated to hyperspectral reflectance (Δλ = 1 nm, 400–700 nm) for each pixel. The interpolated spectra were integrated with the CIE 1931 standard color matching functions [48] to produce the X, Y and Z color primaries and then converted to the standard RGB color table for visual display as a three-channel image file. The X, Y, and Z color primaries were converted to a standard RGB image following the established linear transformation [49].

In this paper, we also examine visible band reflectance data from the GOES-ABI sensor. Ocean color is not an intended application for this sensor. A method to convolve GOES-ABI data with dedicated ocean-color-sensor information is described in Jolliff et al. [21]. Preliminary GOES-ABI Level 1B conus data were obtained from the NOAA Comprehensive Large-Array Data Stewardship System (CLASS) [50]. The ABI data were reconfigured for AOPS processing from Level-1 to Level-2 to obtain the surface reflectance product, as was done for the OLCI and VIIRS data. This surface reflectance product includes most of the standard atmospheric correction but falls short of the aerosol correction. As shown in Jolliff et al. [21], surface reflectance is sometimes sufficient for coastal ocean feature tracking.

The color-enhanced ABI products were constructed from a blend of ABI bands 1 and 2. The 1 km resolution band 1 resolution was mapped to 500 m using a simple left-to-right iterative fill for blended band products. The Supplemental Animations (S1 and S2) show the color-enhanced ABI images at 5 min increments over the LTS study area. Animation S1 is the LTS shelf-wide view, and Animation S2 is focused on the area of frontal movement analyzed herein.

GOES-ABI data are collected at 5 min increments. In order to estimate the velocity of the optical front (described in Section 3. Results), a zonal transect across the apparent position of the front was extracted from the GOES-ABI band 1 reflectance data. Optical front position (or edge detection) was estimated at subpixel resolution by fitting a sigmoid blurred step model to the cross-front reflectance profile using nonlinear least squares fitting and identifying the fitted inflection point as the front location. Function fitting is an established approach for subpixel edge localization [51,52]. The purpose of this technique was to utilize the high-temporal-resolution GOS-ABI data for optical front detection but overcome the 1 km horizontal resolution of ABI band 1.

Results from the American Seas Navy Coastal Ocean Model (AmSeas-NCOM) application [53] were used to examine the physical oceanographic processes occurring during the times coincident with remote sensing data. The 3-hourly AmSeas-NCOM results (1/36°, approximately 3 km horizontal resolution) are provided by the Navy’s Fleet Numerical Meteorology and Oceanography Center and are available to the public through NOAA’s National Centers for Environmental Information. AmSeas-NCOM is a data assimilative ocean model that provides 3D analysis fields for ocean temperature, salinity, and currents. The daily analysis field (0:00 UTC) and the subsequent 3-hourly forecast fields (3:00 to 21:00 UTC) were used in this study. Density values used herein were calculated as a function of simulated salinity and in situ temperature using the UNESCO equation of state [54], and then 1000 kg m^−3^ was subtracted (indicated as σ_t_) from the density value. Where mixed layer depth (MLD) is shown, it was calculated based on the first estimated depth where the density difference from the surface value exceeded 0.03 kg m^−3^ [55]. The MLD reference depth was taken as the surface value in order to indicate where the surface stratification was impacted by diurnal heating. Archived AmSeas-NCOM results are stored at standard reference depths (2 m increments from surface to 12 m, 5 m increments from 15 to 60 m, and 10 m increments to 100 m). Linear interpolation was used to examine the results at 1 m depth increments.

## 3. Results

**Figure 2:**
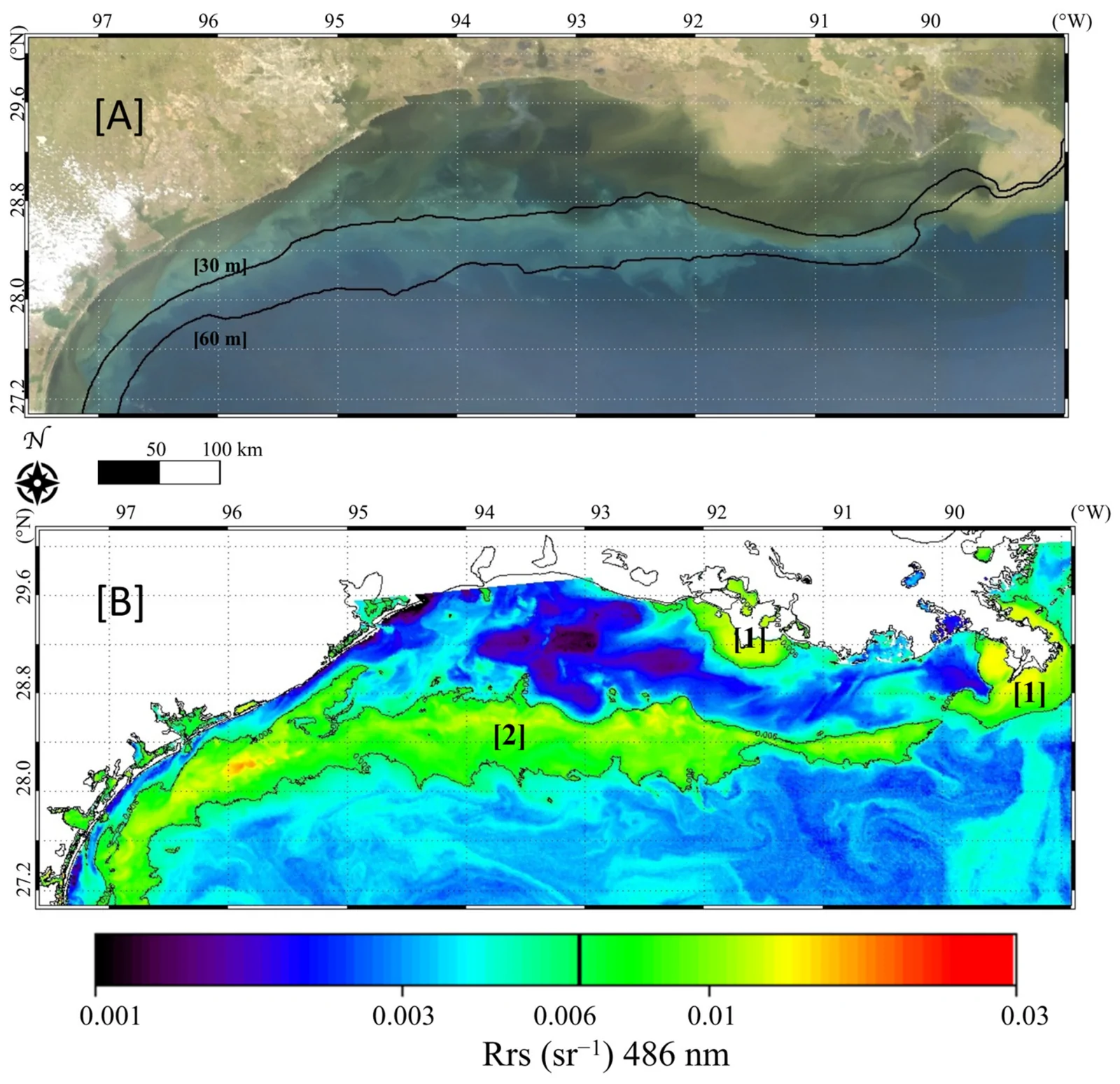
(**A**) True color image based on VIIRS/SNPP visible bands over the LTS region. (**B**) VIIRS/SNPP remote sensing reflectance (Rrs, sr^−1^) for 21 March 2019.

The VIIRS SNPP true color image captured on 21 March 2019 over the LTS (Figure 2A) depicts a nearly shelf-wide, offshore brightness feature extending for approximately 590 km from west to east (28.4° N, ~96.0° to 90.0° W). It is distinct from the features immediately adjacent to the coastline. This apparent brightness feature also extends farther southwestward where the LTS shelf narrows and bends towards the south. Across much of the west to east extent, the feature appears largely confined to overlying the 30 to 60 m isobathymetric contours (Figure 2A). Surface remote sensing reflectance (Rrs) values (486 nm; Figure 2B) enclosed by the 0.006 sr^−1^ contour interval occupy two distinct areas: (1) areas near the Mississippi and Atchafalaya (MA) outflow, and (2) the aforementioned brightness zone extending across the LTS centered approximately along ~28.4° N. Where the portion of the LTS shelf is at its widest extent (~93.15° W) the brightness zone is ~70 km distant from the coastline.

Although the single channel Rrs values (486 nm) are elevated in both areas, the true color contrasts indicate differences in the spectral reflectance signals that are, in turn, likely indicative of different optically active constituents (OACs) in the surface waters. The MA outflow regions (area 1) are often dominated by chromophoric dissolved organic matter (CDOM) as well as suspended organic and inorganic particulates [56]. The OACs giving rise to the other brightness pattern (area 2) are not as well documented. One possibility is very fine silts or calcium carbonate mud slurries that are resuspended from the bottom [57,58]. This is consistent with similar optical signals observed elsewhere in the region during periods of vertical hydrodynamic instability [22,23].

**Figure 3:**
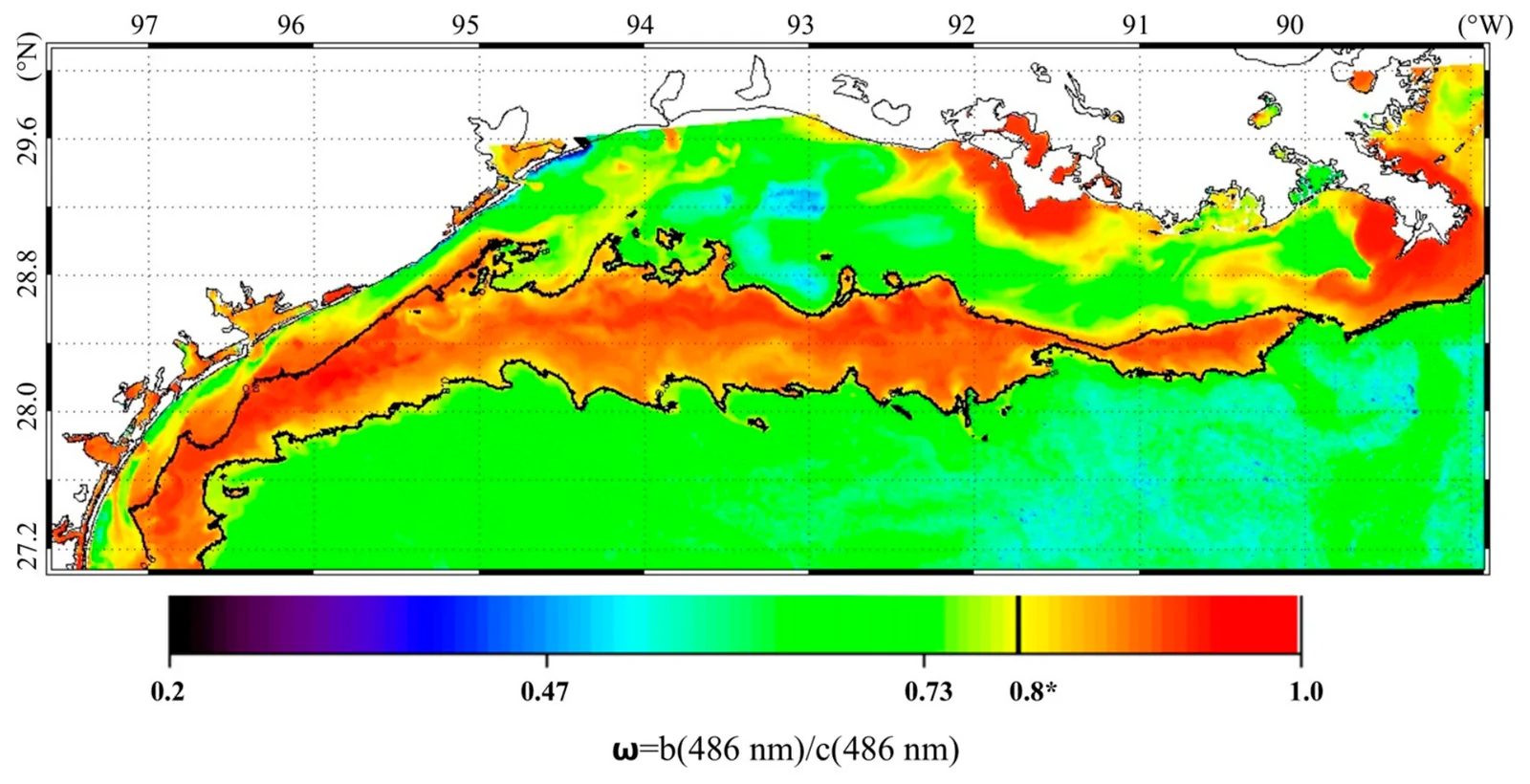
VIIRS SNPP on 21 March 2019 contouring the scattering albedo (486 nm). The contour line indicates where the scattering albedo > 0.8 and the reflectance ratio (as described in the text) exceeds 0.5.

In both areas, the elevated Rrs is consistent with enhanced particle scattering; we use ‘turbidity’ here as a descriptive term for these high-reflectance signals. To distinguish the two observed spectral-reflectance classes, we examine the retrieved scattering albedo and relative blue-band reflectance (Figure 3). The scattering albedo is simply the ratio of the spectral scattering coefficient to the beam attenuation coefficient [45,47], and it is a direct measure of the relative amount of scattering in the contribution to beam attenuation (the sum of absorption and scattering). The contour line (Figure 3) indicates where the satellite-estimated scattering albedo (486 nm) exceeds 0.8 (scattering is more than 80% of the total attenuation) and the ratio of reflectance at (486 nm) to the sum of reflectance at 486 and 551 nm exceeds 0.5. The increased reflectance in the blue (towards 486 nm) corresponds to the true color signal (Figure 2A) where the offshore turbidity is shifted towards the blue, and in contrast, the river outflow turbidity is shifted towards the red. A previous study identified this type of offshore turbidity feature as “blue-shifted” turbidity [59].

**Figure 4:**
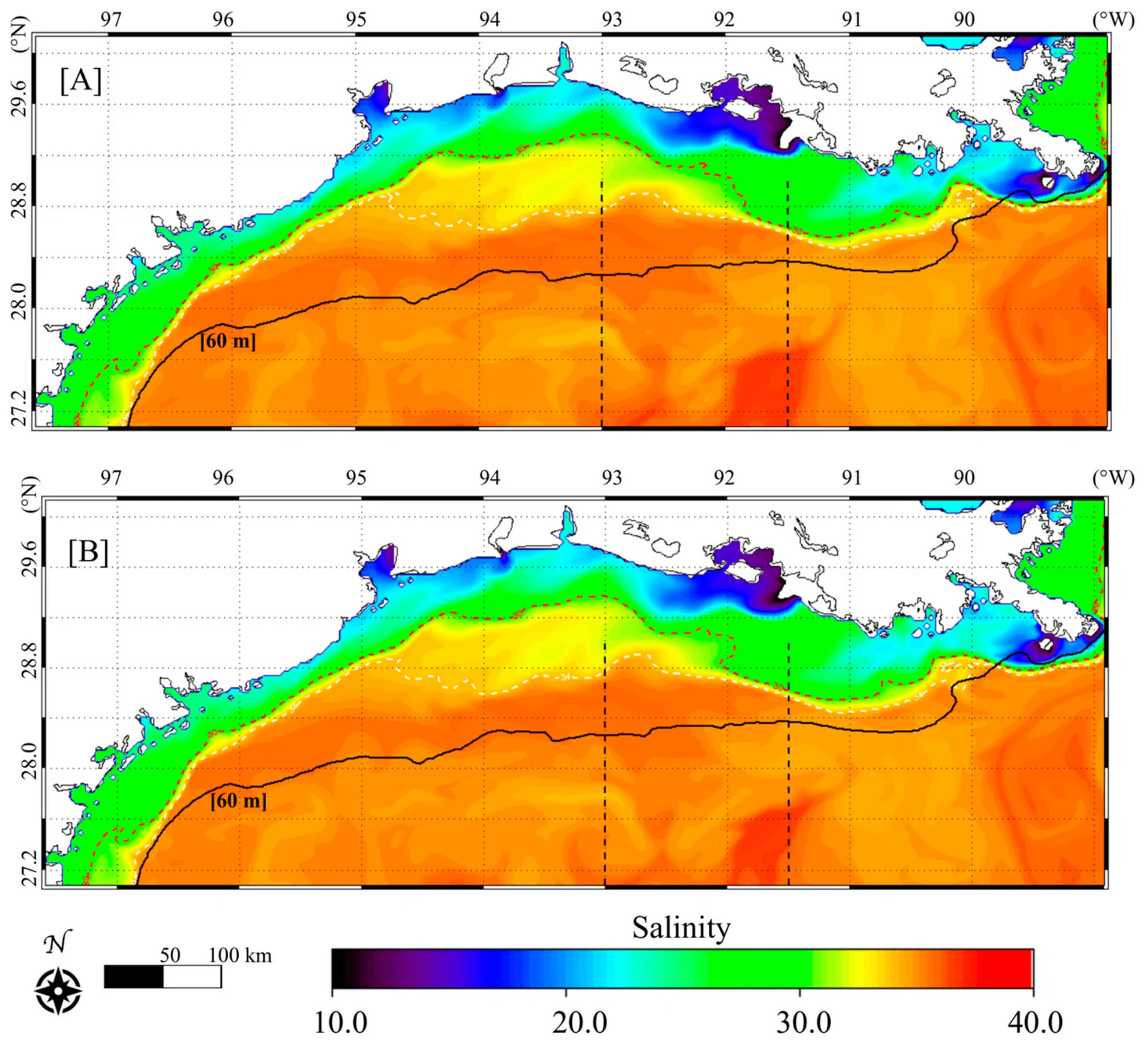
NCOM surface salinity results (AmSeas-NCOM) for (**A**) UTC 06:00 and (**B**) UTC 21:00 on 21 March 2019. The white dashed line is the surface salinity (34 PSU) and red dashed line for 30 (PSU) isohalines. The black dashed lines are cross-shelf transects at 93 and 91.5° W longitude that are shown in subsequent figures. The solid black line is the 60 m bathymetry contour from the DBDB2 dataset.

The freshwater influence across the LTS is indicated in NCOM results (Figure 4). The 34 surface salinity isopleths (isohalines: white dashed lines, Figure 4) are used to delineate shelf water masses from those of the open ocean. It is also roughly indicative of a frontal boundary that corresponds to the location of the blue-shifted turbidity features that extend across the shelf. The major freshwater outflows from the MA generally propagate westward, consistent with Coriolis forcing (to the right of the outflow direction) as well as previous observations of coastal currents over the LTS [60]. This westward flow establishes a cross-shelf surface salinity gradient across the LTS.

The elevated areas (2) of offshore turbidity in the satellite data also appear to correspond to the areas in the model between the 34 isohaline and the 60 m isobath (Figure 2 and Figure 4). There is also an area of apparent bifurcation in the simulated 30 and 34 isohalines: in the east the isohalines are close together until around 91.8 to 92° W where they separate (Figure 4). This is the area where the bathymetry also bifurcates as the shelf becomes wider: the 30 m isobath continues west while the 10 m isobath follows the northern bend of the coastline (Figure 1B). In the area where the 30 and 34 isohalines separate, there also appears to be some simulated westward movement of the 30 isohaline from 06:00 UTC (Figure 4A) to 21:00 UTC (Figure 4B).

**Figure 5:**
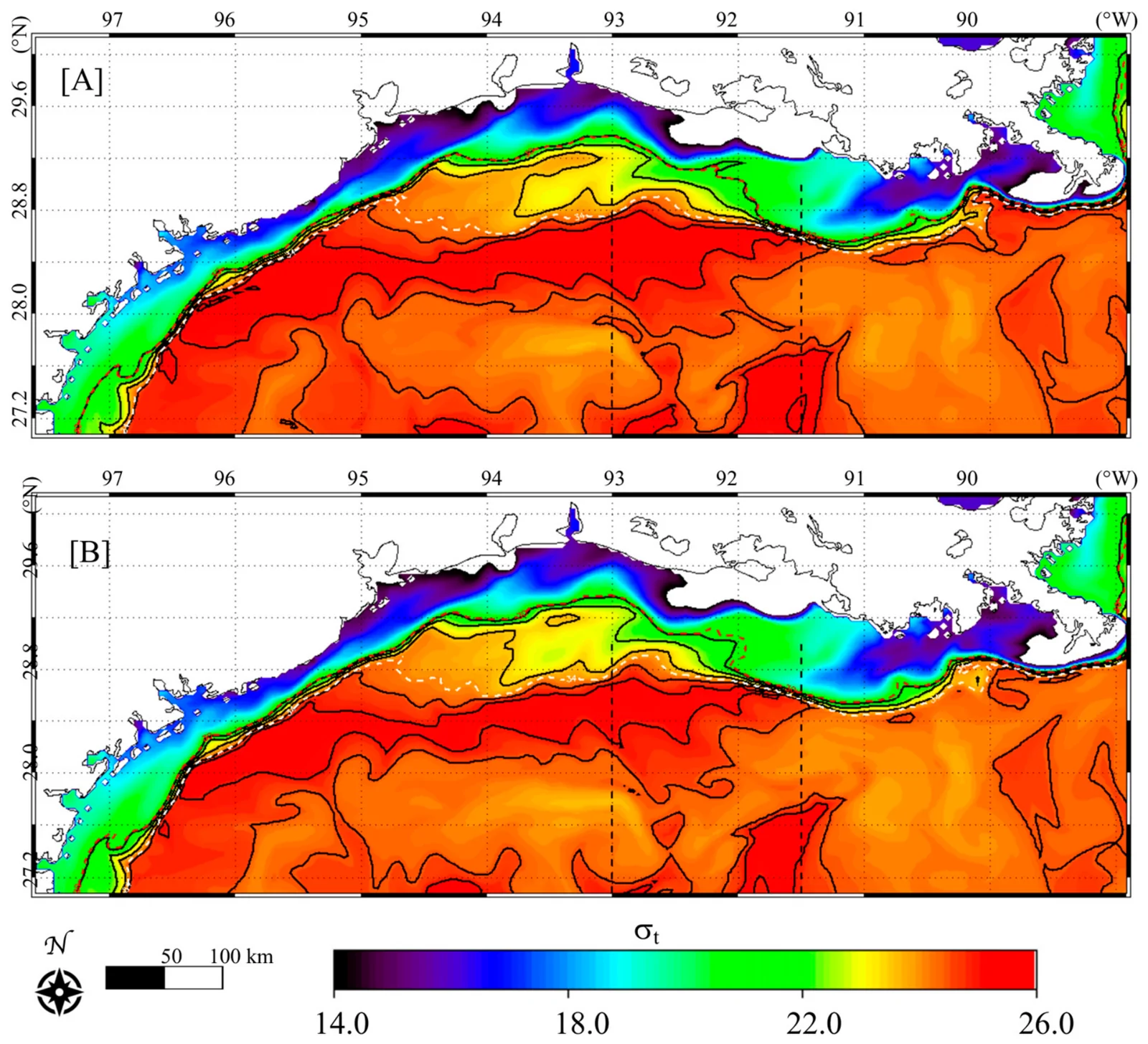
Surface density for (**A**) 06:00 and (**B**) 21:00 UTC from Am-Seas NCOM results for 21 March 2019. The white dashed line is the surface salinity (34 isohaline) and red dashed line for 30. The black dashed lines are cross-shelf transects at 93° and 91.5° W longitude that are shown in subsequent figures.

Frontal boundaries, however, are more properly identified by a horizontal gradient in surface density, which is evident by the apparent reduction in horizontal distance between constant density isopleths (isopycnals) in the simulated density fields (Figure 5). In the eastern LTS (south of Atchafalaya Bay, from ~91.0° to 91.8° W), the isopycnals are very tightly packed across a gradient of ~22 to 25 kg m^−3^ (Figure 5), also where the 30 and 34 isohalines converge (Figure 4). The front appears to also mark the boundary between the offshore turbidity water mass identified in the true color images and the reflectance values (Figure 2 and Figure 3) and shelf waters influenced by freshwater outflow. As in Figure 4, between 06:00 and 21:00 UTC, the modeled density contours shift westward near the isopycnal bifurcation (92° W and 28.8° N; Figure 5A,B).

**Figure 6:**
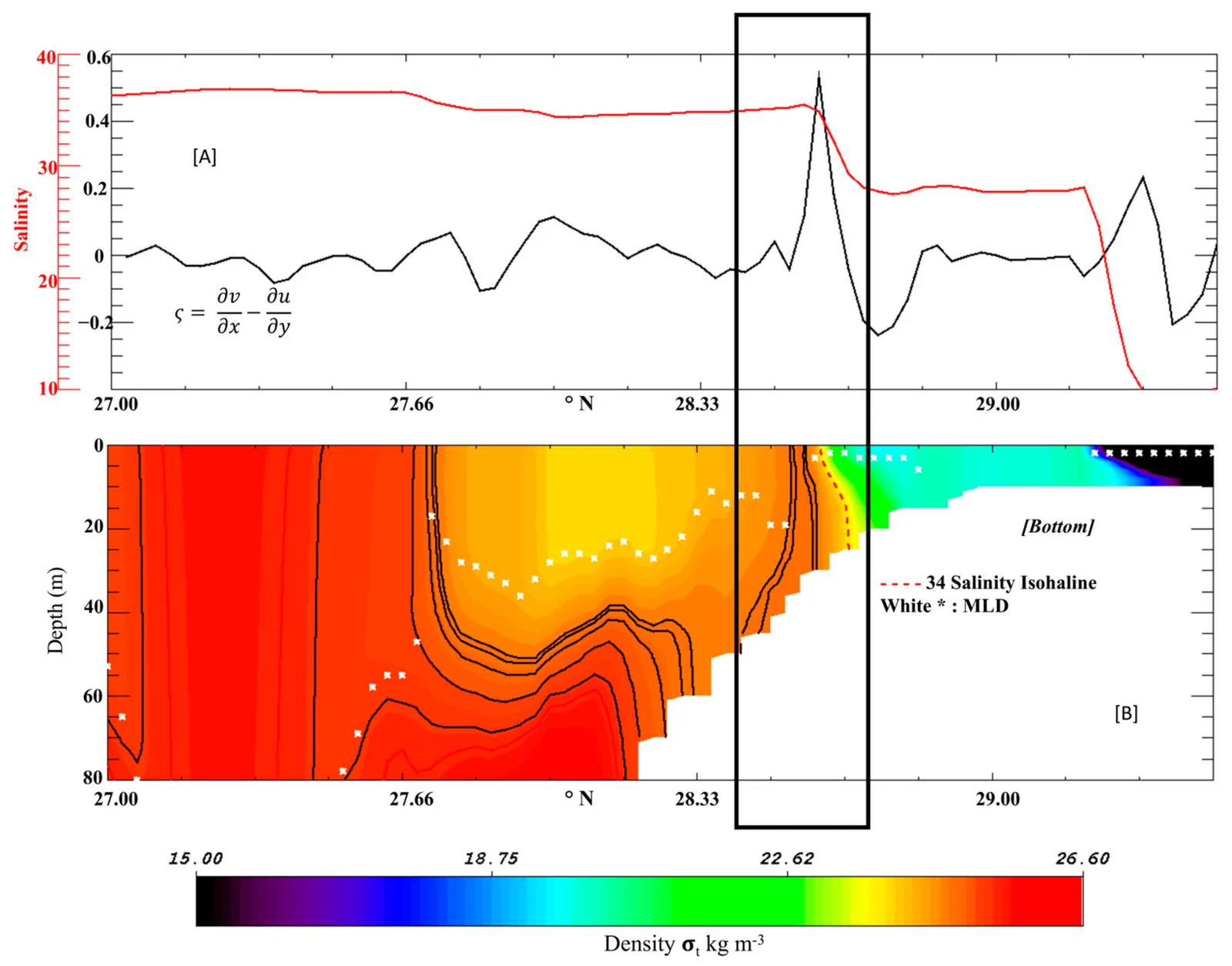
Results from Am-Seas NCOM for 21 March (06:00 UTC, 91.5° W): (**A**) relative vorticity along the meridional transect (black line) and surface salinity (red line); (**B**) NCOM density contours along the cross-shelf transect, the white dashed line in the mixed layer depth and the red dashed line is the 34 isohaline. The boxed area indicates the position of the front.

A cross-shelf transect through the NCOM results for 21 March (Figure 6) illustrates the potential relationships between the density field and the optical signals observed (the transect corresponds to the dashed black lines in Figure 4 and Figure 5). The simulated cross-shelf density distribution reveals the frontal boundary at approximately the 30 m shelf depth: the nearshore densities are much lower due to the lower salinities and just on the seaward side of the boundary (at the 34 isohaline: dashed red line Figure 6B) isopycnals are vertical before becoming horizontal in deeper waters. The relative vorticity is estimated from the NCOM surface velocities (Figure 6A). There is a sharp positive peak in the relative vorticity on the seaward side of the front. There, constant density isopleths (isopycnals) extend from the surface to the bottom. This spatial correspondence is consistent with, but does not explicitly demonstrate, a potential contribution from resuspended near-bottom materials to the optical signals detected.

The positive peak in the simulated relative vorticity along the seaward side of the front appears to mark a transition from stratified inner shelf waters to a more well-mixed offshore zone. The vertically oriented isopycnals indicate that the modeled density front spans much of the water column between approximately 40 and 30 m depth. This simulated pattern, however, is not consistent throughout the diurnal cycle. Superimposed on this frontal boundary is the seasonal transition from frequent episodes of vertical mixing to seasonally persistent horizontal stratification, referred to as the spring transition [61,62,63] for mid-latitude shelves.

**Figure 7:**
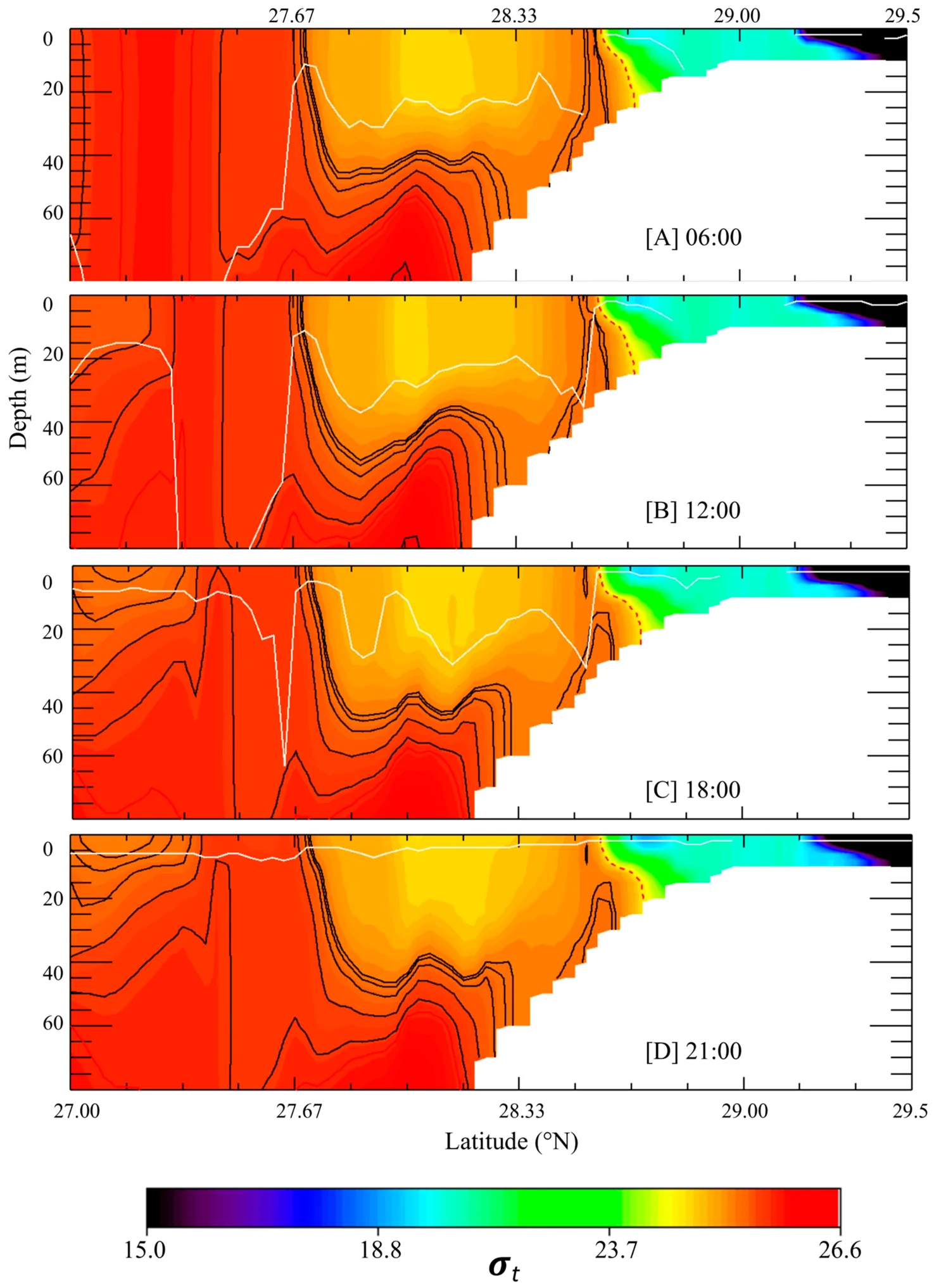
NCOM cross-shelf simulated density transects (91.5° W) on 21 March for (**A**) 06:00 UTC; (**B**) 12:00 UTC; (**C**) 18:00 UTC; and (**D**) 21:00 UTC. The white line is the mixed layer depth and the red dashed line is the 34 isohaline.

Time series of the simulated cross-shelf density distribution indicate a progressive weakening of the isopycnals structure along the seaward side of the front (Figure 7). In addition, the 34 isohaline becomes progressively deformed from vertical (Figure 7A) towards more horizontal (Figure 7D). The mixed layer depth across the shelf also transitions from highly variable across the front to nearly uniform at less than 10 m depth. The times are consistent with a progressive diurnal warming, and this warming is simultaneous to the deformation and surface movement of the frontal boundary.

**Figure 8:**
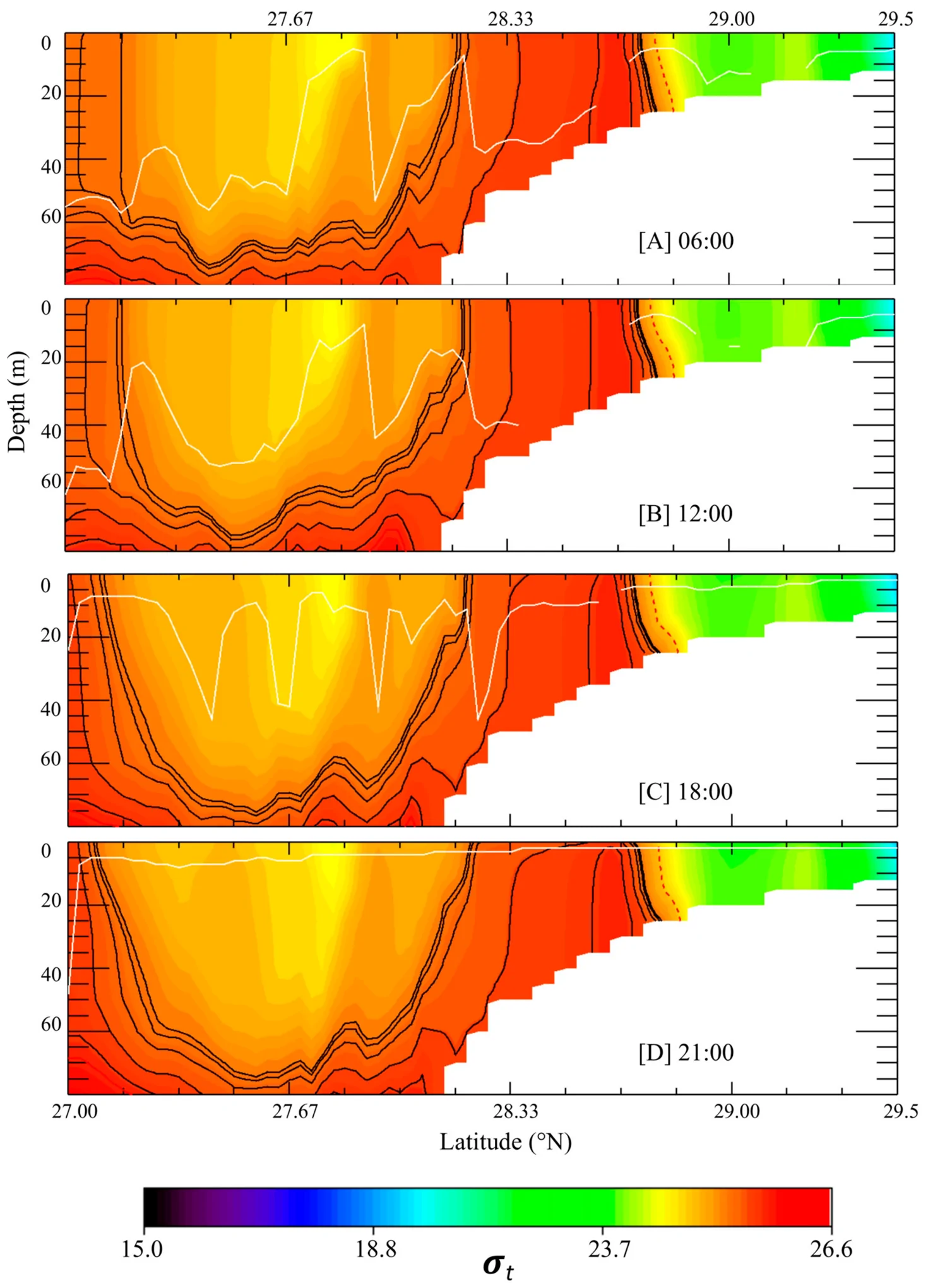
NCOM cross-shelf simulated density transects (93.0° W) on 21 March for (**A**) 06:00 UTC; (**B**) 12:00 UTC; (**C**) 18:00 UTC; and (**D**) 21:00 UTC. The white line is the mixed layer depth and the red dashed line is the 34-salinity contour interval.

A similar transect through the model results farther west (93° W) shows a very similar pattern (Figure 8). The dynamics there are slightly altered: a very broad area (~28.3–28.8° N) shows isopycnals extending from the surface to bottom (Figure 8A,B). As the day progresses, however, a diurnal surface warming appears to establish a very shallow mixed layer across the shelf front (Figure 8C,D). The main shelf front (the dashed red line for the 34 isohaline at 28.8° N) does not appear to move in the cross-shelf direction. This is in contrast to the apparent simulated movement of the front for the transect farther east (Figure 7C,D).

**Figure 9:**
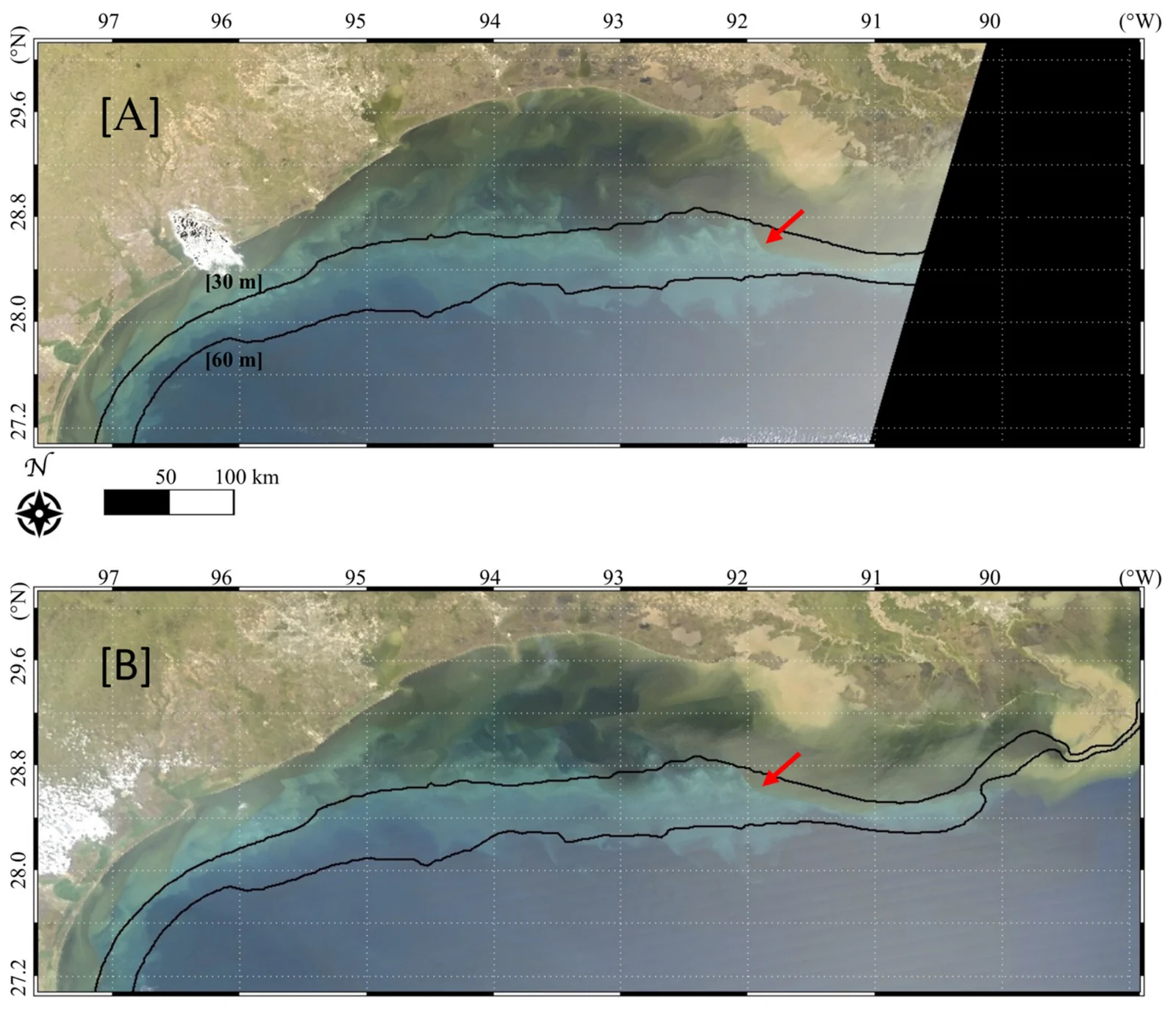
True color images 21 March (2019): (**A**) OLCI (Sentinel 3B), granule capture 16:34:45 UTC; (**B**) VIIRS NOAA-20, granule capture 19:00:50. The red arrow indicates movement of the front over the blue-shifted turbidity.

**Figure 10:**
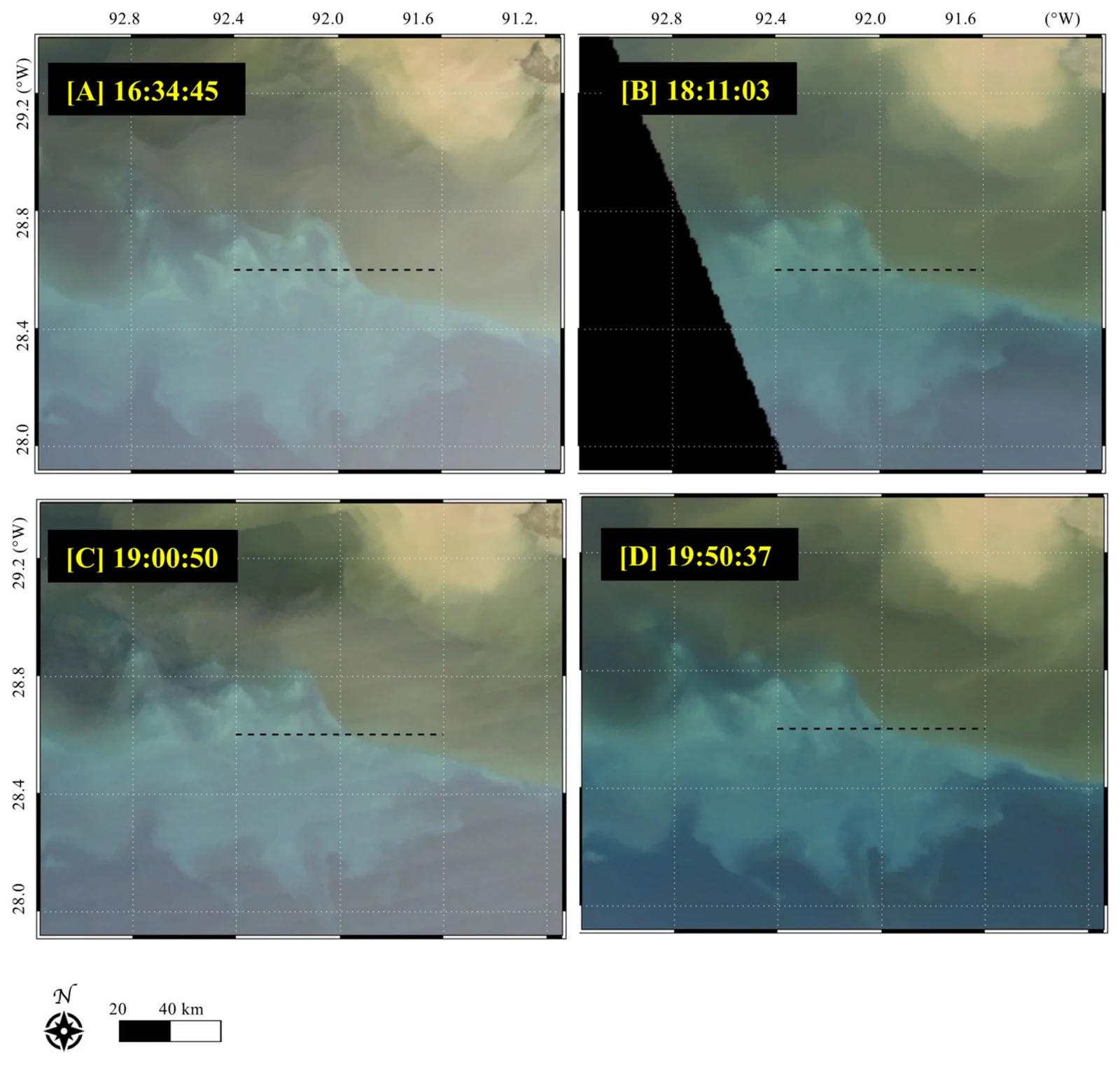
Isolated view of the front movement using true color images on 21 March (2019): (**A**) OLCI (Sentinel 3B), capture 16:34:45 UTC; (**B**) VIIRS SNPP, 18:11:03 UTC; (**C**) VIIRS NOAA-20, 19:00:50 UTC; (**D**) VIIRS SNPP 19:50:37 UTC.

The NCOM simulated dynamics appear to be qualitatively consistent with patterns revealed from multi-sensor ocean color images of the LTS (Figure 9). The optical frontal boundary immediately south of Atchafalaya Bay (red arrow, Figure 9) appears to progress towards the west to southwest between the time of the OLCI image (Figure 9A; ~10:34 local CST) and the VIIRS NOAA-20 image (Figure 9B; ~13:00 local CST) by approximately 8 km. The inferred optical front movement is conspicuous when all four of the ocean-color-sensor-based true color images are compared (Figure 10). The true color images were constructed from the OLCI and VIIRS instrument data on four satellite platforms (Sentinel 3B, SNPP and NOAA-20). The morning OLCI pass indicates the initial position of the optical front is east of 92° W, and the final image (19:50 UTC) shows much of the optical front is west of 92° W (Figure 10A,D). The movement, however, is largely to the west and in the LTS area where both the bathymetric contours and the NCOM simulated surface isopycnals diverge.

**Figure 11:**
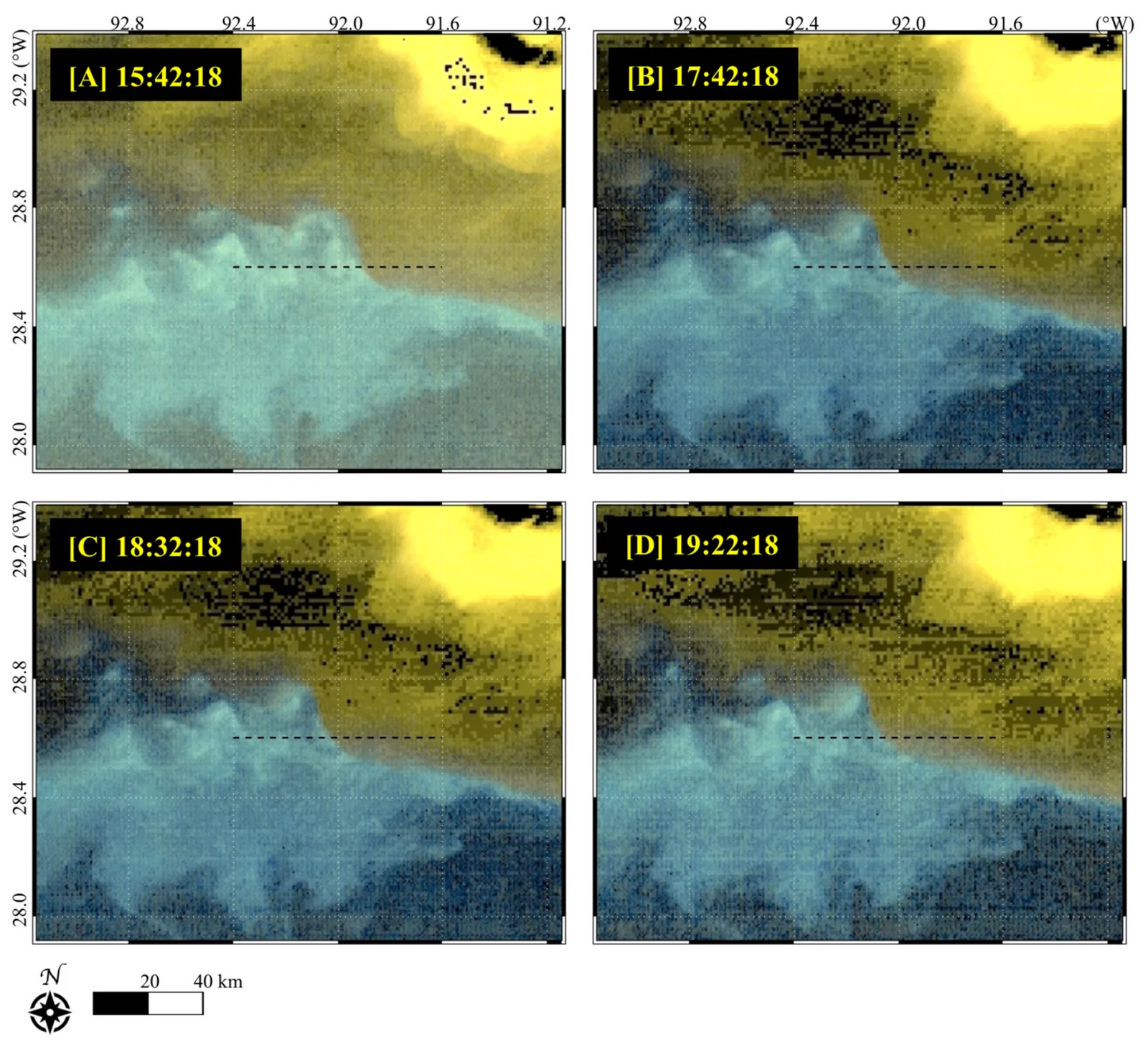
Color-enhanced image sequences based on GOES ABI data for 21 March: (**A**) 15:42:18 UTC; (**B**) 17:42:18 UTC; (**C**) 18:32:18 UTC; (**D**) 19:22:18 UTC.

High-temporal-resolution image sequences from color-enhanced GOES ABI data (Supplemental Animations S1 and S2) provide more qualitative detail on the kinematics of the apparent optical front movement. In the shelf-wide LTS image sequence (Animation S1) the westward movement of the optical front is conspicuous because true color gradients do not appear to be moving as rapidly elsewhere across the LTS. A closeup image sequence of the optical front movement (Animation S2), as resolved by color-enhanced ABI images (Figure 11), corroborates what is shown in the ocean color sensor images: the optical front appears to be east of 92° W in the morning, local time, and then largely west of that longitude ~4 h later (Figure 11).

**Figure 12:**
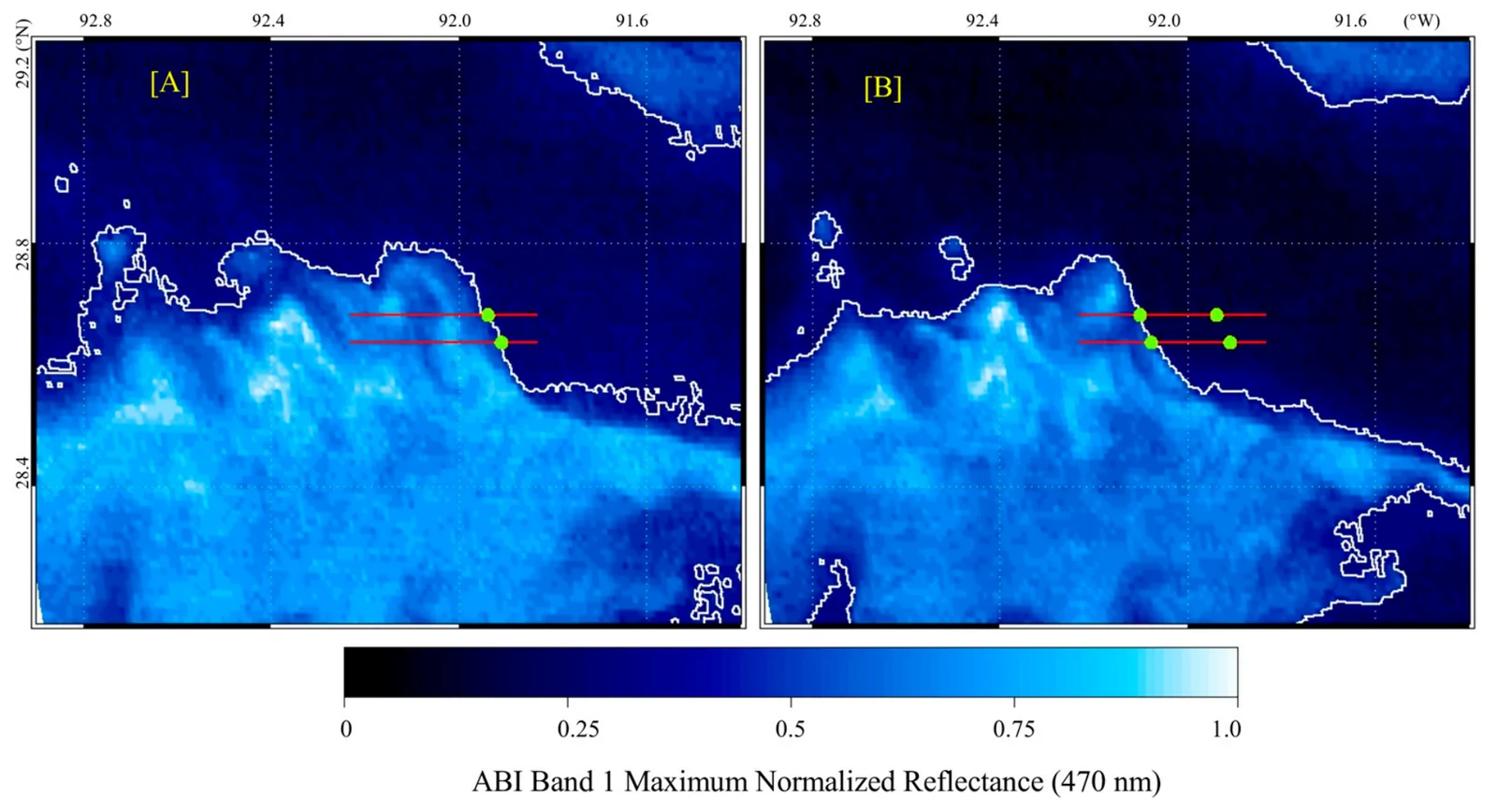
Normalized surface reflectance ABI band 1 for (**A**) 15:07 and (**B**) 20:07. Transects (red line) were used to estimate zonal velocities, and the green dots indicate the estimated front positions. The zonal transects are 28.66° and 28.61° N.

For an initial quantitative examination of the ABI sequence, band 1 (470 nm) reflectance was selected because it provided the clearly discernable contrast across the putative optical front. The processed ABI data (Level 2) were not corrected for bidirectional reflectance distribution function (BRDF) variance in the retrieved reflectance signal; this required that for each datafile, the ABI band 1 reflectance values were normalized by the maximum value in the scene. Initially, the optical edge position was operationally defined as the location where normalized reflectance fell below 40% along each zonal transect (Figure 12A), although sensitivity to the threshold and normalization is a significant potential source of positional uncertainty (Figure 12A). Between the initial (15:07) and the final images shown (20:07) UTC, the analysis implies a mean optical edge zonal velocity of approximately −0.90 m s^−1^ (Figure 12B) for both transects.

**Figure 13:**
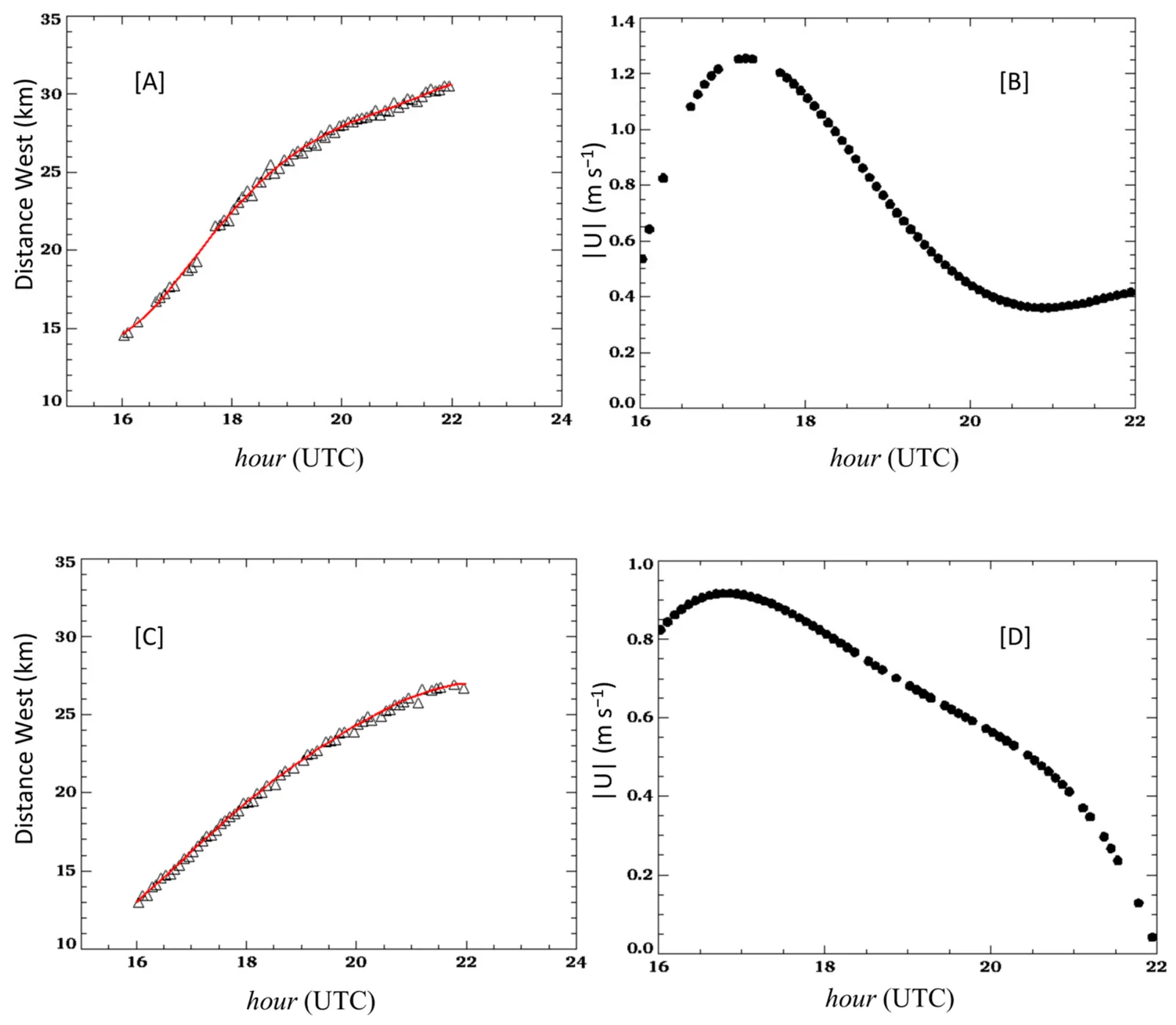
The edge-detection algorithm applied to ABI band 1 for the transect along 28.68° N: resulting positions along the transect (**A**), the red line is a 5th-order polynomial fit to the estimated edge locations as a function of time. The first derivative of the polynomial fitted in (**A**) is shown as the optical front velocity curve in (**B**). The same results are shown for position (**C**) and derived velocity (**D**) for a lower transect (28.61° N).

Evaluating the normalized reflectance contours is a rudimentary form of edge detection. To exploit the 5 min ABI data, we turned to more formal edge detection methods [51]. Here, reflectance data were not normalized to avoid any potential artifacts from excessively bright pixels. The ABI time series of resolved edge (optical front) locations are shown for two transects across the front (Figure 13). Excessive positional outliers, very common at the beginning and end of the daily record due to BRDF, were excluded from the analysis. The two positional curves and derived velocities reveal similar patterns: highest velocities between 16:00 and 18:00 UTC, with some edge apparent deceleration thereafter. Although angular effects from the sensor to solar zenith geometry cannot be ruled out as contributing to the apparent variation in the position and velocity curves, the data indicate more significant frontal boundary movement occurring before the polar-orbiting observations tend to be clustered (18:00–20:00 UTC), with Sentinel 3A/B being the exception.

**Figure 14:**
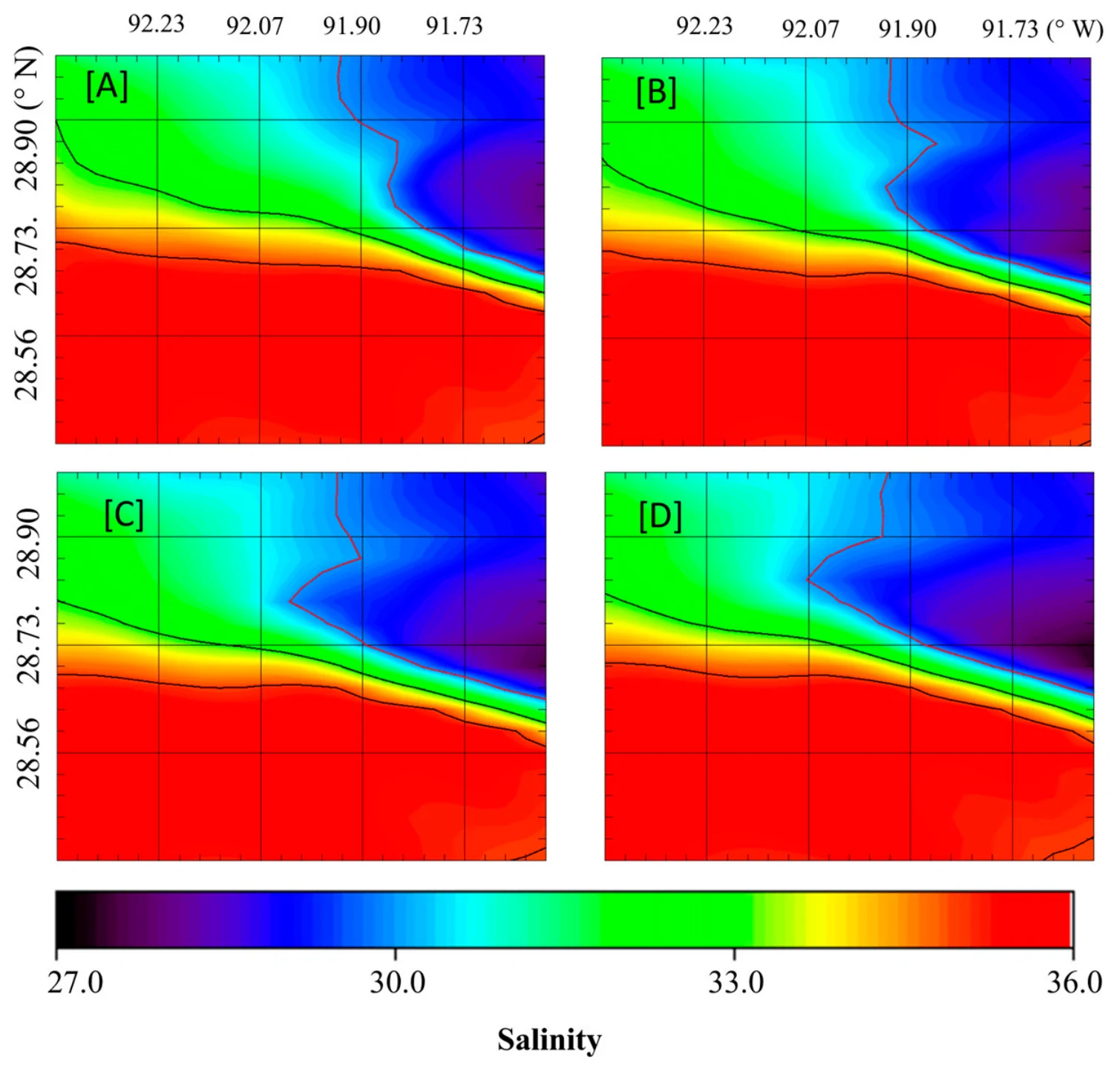
NCOM surface salinity results shown for (**A**) 06:00 UTC, (**B**) 12:00 UTC, (**C**) 18:00 UTC, and (**D**) 21:00 UTC on 21 March. The red contour line is the 30 isohaline, and the black lines are 33 and 35 isohaline.

A detailed examination of the NCOM results indicates a similarly oriented density front, dominated by the salinity gradient near ~92° W (Figure 14). Qualitatively, the orientation of the front bifurcation in NCOM is similar to the one estimated from satellite data—a front oriented largely east to west until about ~92° W where the orientation turns more north to south between roughly 28.5° to 28.9° N (Figure 12 and Figure 14). There is a bifurcation in the surface isohalines there as well, with the 34 and 35 isohalines continuing west and the 30 isohaline veering north. It is the north-to-south-oriented side of the front that appears to propagate westward in the satellite data, although there is no verification of the surface salinities from these satellite data alone. The westward progression of the modeled north–south 30 isohaline is qualitatively similar to the satellite optical front displacement. Although the variables and space/time resolutions differ, there is an apparent westward movement of the 30 isohaline between 06:00 (Figure 14A) and 21:00 UTC (Figure 14D).

**Figure 15:**
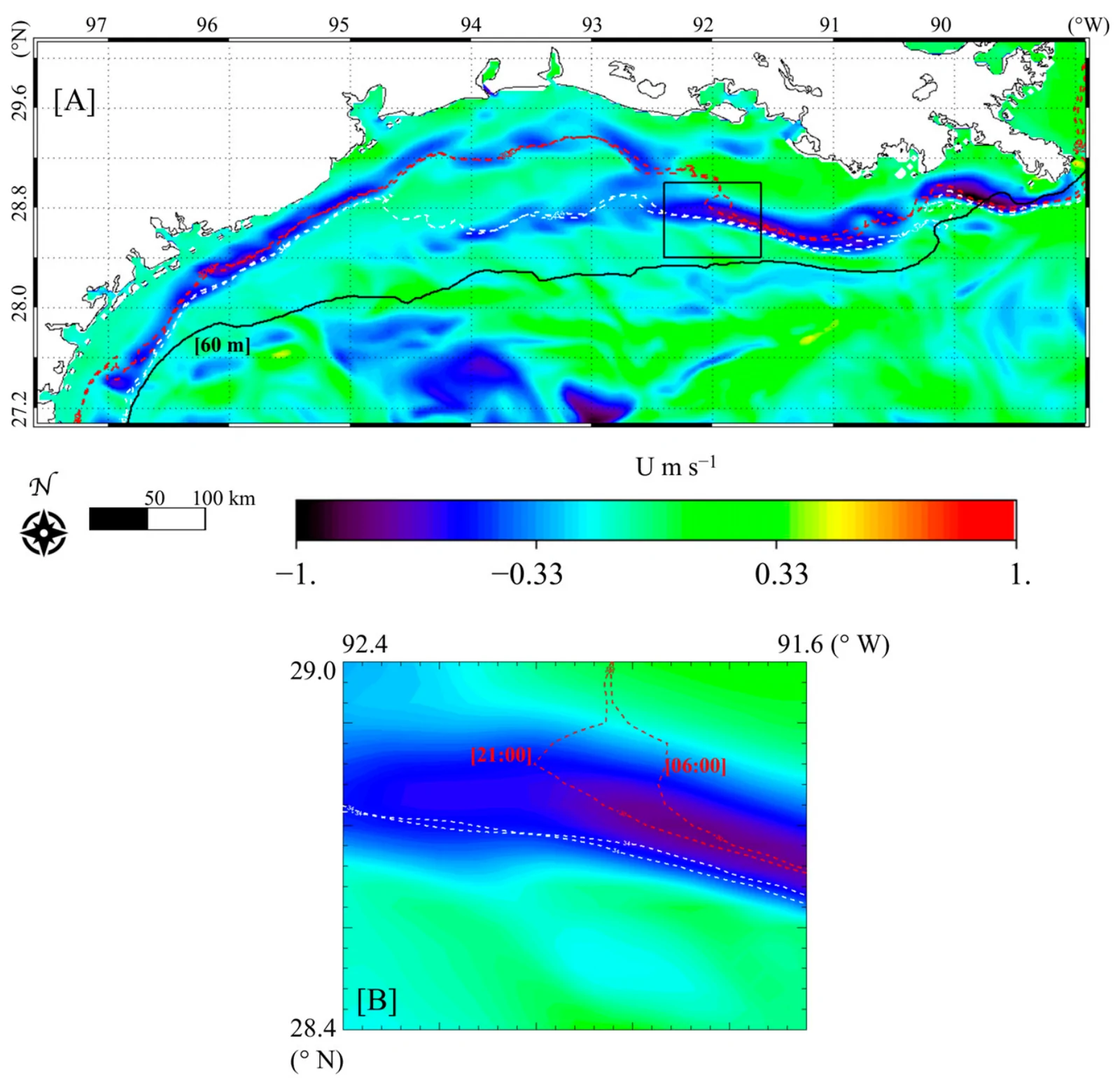
Regional NCOM results for surface zonal velocities (U, m s^−1^) (**A**); and a close-up detail of same in the boxed area (**B**) from 21:00 UTC. The solid line is the 30 isohaline (red) and the 34 isohaline (white) at 06:00 UTC and 21:00 UTC.

The similar movement of the front implied by the ocean color image sequences and NCOM simulation provide an opportunity to evaluate candidate physical mechanisms. The westward current flow and the spatial orientation of the front generally follow the isobathymetric contours, consistent with previous descriptions of the LTS circulation [42]. A regional view of surface zonal (U) velocities (Figure 15A) shows increased zonal flow to the west near the main frontal boundaries, as indicated by the 30 and 34 isohalines. Note that the westward velocities appear more intense where the 30 and 34 isohalines are together, and appear comparatively weakened when isohalines are separated.

The simulated westward velocity along the front is consistent with the combined effects of buoyancy-driven circulation and wind-driven transport expected after a cold-front passage [42,64]. Under the northerly wind regime, both forcing mechanisms act synergistically to intensify zonal velocities at the front towards the west [65], and a very strong, narrow current (baroclinic jet [66]) tends to develop on the front edge [67]. Within the boxed area (Figure 15B), where there is a bifurcation in both the 30 and 34 isohalines, the most intense zonal surface velocities occur before the isohalines divergence.

**Figure 16:**
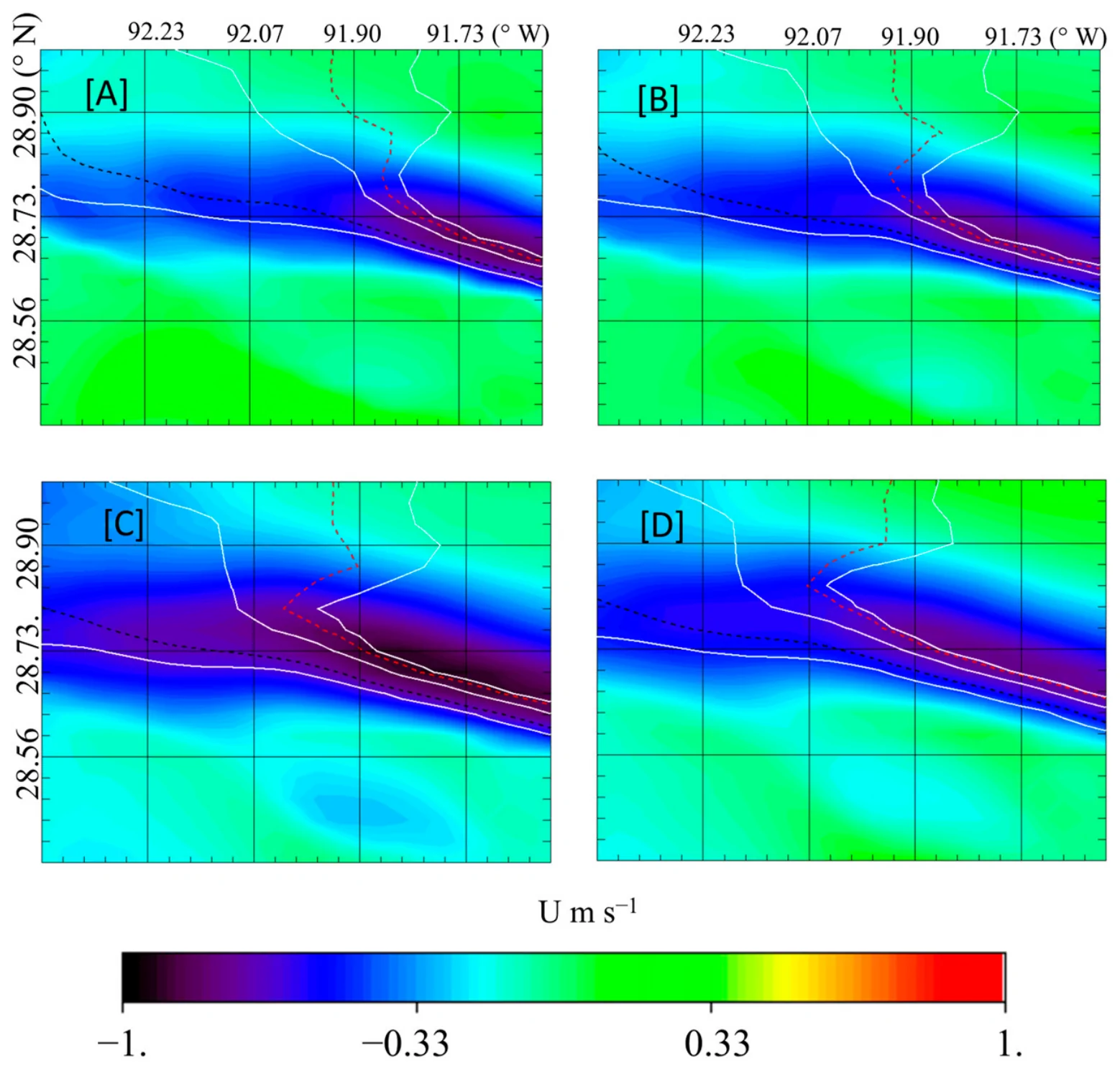
NCOM surface velocity results shown in (**A**) 06:00 UTC, (**B**) 12:00 UTC, (**C**) 18:00 UTC, and (**D**) 21:00 UTC on 21 March. The red dashed contour line is the 30 isohaline, and the black dashed line is the 34 isohaline. The white contour lines are the 21, 22, and 24 isopycnals (σ_t_, kg m^−3^), from right to left.

The colocation of the strongest density gradients with elevated westward velocities is consistent with combined buoyancy-driven and wind-driven transport (Figure 15). At the beginning of 21 March, the maximum velocities occur where the surface isopycnals are closer together (Figure 16A), east of 91.9° W. Twelve hours later, the maximum velocities, the tightly clustered isopycnals, and the point of isohaline bifurcation are all protruding west of 91.9° W (Figure 16C). The point of bifurcation is being pushed westward as the low-salinity plume is effectively advected to the west, the most intense westward currents occurring where the isohalines (and isopycnals) are closest together along the edge of the front. This is to be expected since the portion of the flow driven by the density gradient (baroclinic) is strongest where the gradient is largest.

**Figure 17:**
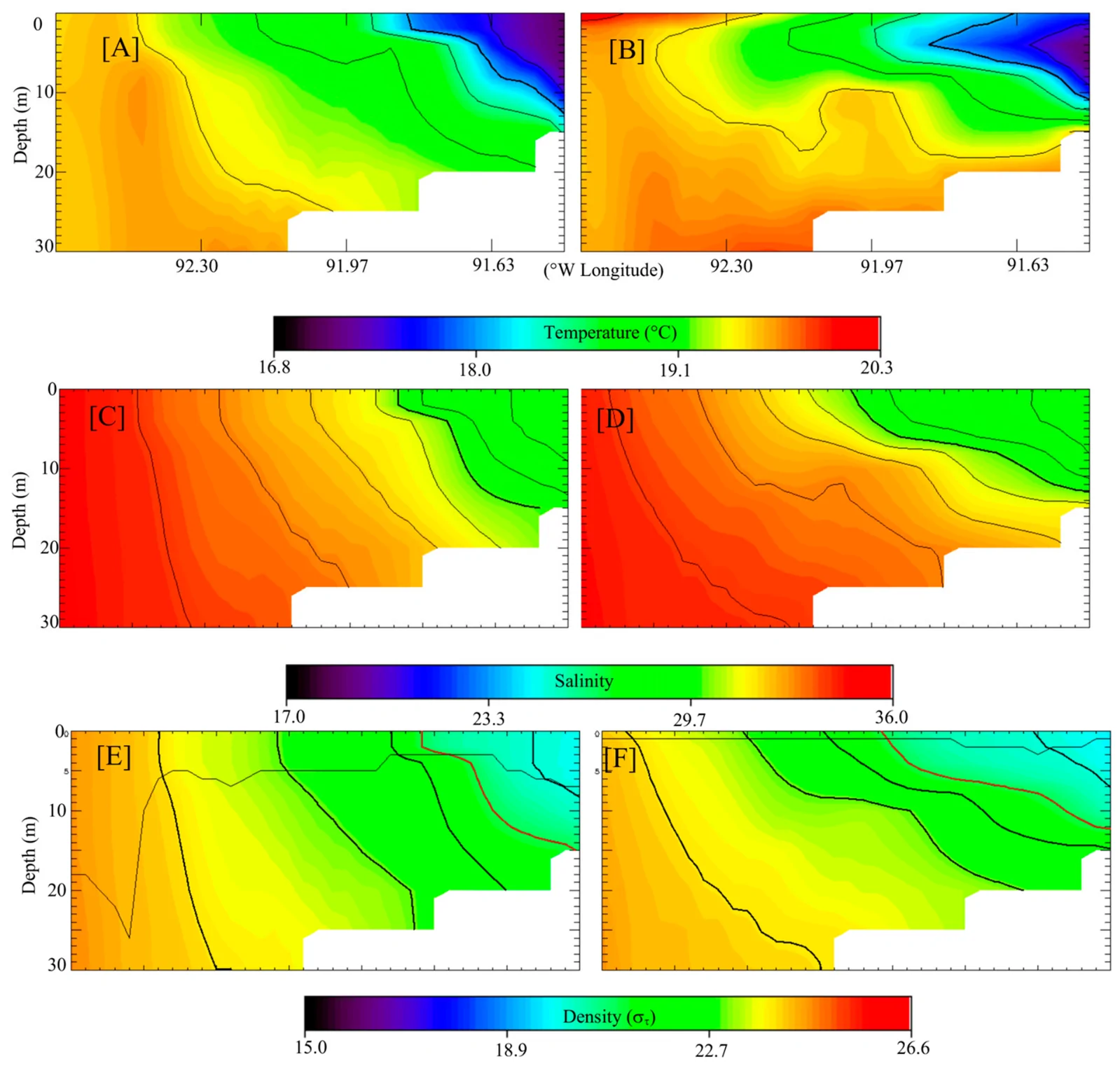
NCOM results for a zonal transect along 28.83° N latitude (between 92.83 to 91.17° W) for temperature (**A**) 06:00 UTC, (**B**) 21:00 UTC; salinity (**C**) 06:00 UTC, (**D**) 21:00 UTC; density (**E**) 06:00 UTC, (**F**) 21:00 UTC. Thin Black line for (**E**,**F**) is the mixed layer depth calculated from the surface layer.

In addition to the intense coastal jet concentrated along the frontal boundary, the model suggests that the diurnal heating is also contributing to the surface density stratification. A zonal transect through the boxed area shown in Figure 14 and Figure 16 shows the initial model state as the day begins (Figure 17): the temperature isopleths follow the salinity isopleths and are largely vertical along the shelf resulting in the vertical density isopycnal structure (Figure 17A,C,E), with a slight degree of declination towards the west. Some fifteen hours of simulated time later (1500 local time, CST), the upper surface waters (<5 m depth) have warmed significantly (Figure 16B), resulting in a salinity-compensated temperature inversion. Since the lower-salinity waters are initially colder, this creates a temperature inversion compensated by the salinity so that the density profile remains stable. The density contours, however, are transitioning from largely vertical (Figure 17E) to an increased declination to the horizontal (Figure 17F).

The very shallow mixed-layer depth (Figure 17F) suggests the upper water column is continuously stratified. Although significant salinity stratification is present in the initial state (Figure 17E), there is a surface mixed layer in the upper 10 m consistent with convective cooling during nighttime hours. Once the day begins, the solar heating is making a very thin, warm surface layer cap. Increased near-surface stratification would be expected to reduce surface–bottom frictional coupling [68] and could thereby facilitate cross-shelf spreading of the low-salinity surface layer, although this mechanism is not quantified in the present simulation analysis. In other words, the wind stress is more effective at moving the surface plume when and where there is weakening of surface–bottom frictional coupling over the shelf [42,69]. Indeed, in this area where the shelf widens there is an increase in water depth with zonal movement to the west.

## 4. Discussion

The utility of the geostationary ABI data demonstrated herein is primarily the confirmation and examination of the optical boundary movement as the day progresses. Many of the polar-orbiting sensor images tend to cluster around ~18:00–20:00 UTC for the LTS study area, with the OLCI pass on the Sentinel A and B satellites providing the only morning (local time) views. Due to orbital gaps, only one of the Sentinel satellites usually resolves an image of the study area for a given day, and if clouds obscure the scene at the acquisition time, then there will be no morning data available. The utilization of the ABI data permits the analysis to proceed with a comprehensive estimate of the movement throughout the day. For example, 21 March 2019 allowed for both an OLCI morning view as well as two passes from SNPP, which are also not always available. If the OLCI data and the first SNPP pass were not available, the contrast of the remaining two images (Figure 11C,D) would not be sufficient to resolve the apparent motion of the front. It is combined use of ABI with the other polar-orbiting sensors that provided more context and confirmation for apparent feature movements. The ABI-based edge-detection technique provided the optical front velocity estimates over roughly 6 h (Figure 13).

Taken together, the combined analysis of the multi-sensor satellite data and the NCOM simulation point to interrelated and cascading scales of events that give rise to the ocean color signals observed. During the post-CAO northerly winds, NCOM shows intensified westward currents along the modeled density fronts. This pattern is consistent with combined wind-driven transport and freshwater-induced buoyancy circulation [70,71]. NCOM displays positive (cyclonic) relative vorticity on the seaward side of the modeled front, spatially adjacent to the satellite-observed high blue-band reflectivity. In the area where there is a bifurcation in the LTS bathymetry, the spatial orientation of the front becomes orthogonal to the prevailing current and westward advection becomes conspicuous in ocean color image sequences. Simultaneous to this process, the initial ocean heat loss from the CAO event subsides and increasing solar insolation brings about a diurnal warming of the surface layer that reinforces the density stratification. The ability of NCOM to simulate these processes appeared to result in some qualitative similarity between simulated surface isopycnal (and isohaline) movement with the optical front identified in the VIIRS, OLCI, and GOES-ABI sensor records.

Our hypothesis is that the color signature of bottom water ventilation events in the LTS system is a bright white to turquoise turbidity feature that is optically distinguishable with a high scattering albedo and a significant increase in the blue reflectance bands when compared to the shelf waters influenced by the significant freshwater effluent. The identification of the materials responsible for this higher blue reflectivity described herein is speculative and requires verification. Nonetheless, the NCOM results indicate isopycnals extending from near the shelf bottom to the surface along the seaward side of a shelf front are accompanied by an increase in relative vorticity. This suggests the occurrence of rapid vertical water displacements near frontal boundaries that are only now being recognized and further investigated in the LTS system [72,73].

Upwelling along the front would further suggest enhanced biological productivity due to the potential surface ventilation of nutrients. However, phytoplankton pigments tend to diminish blue spectral reflectance via absorption by pigments. Instead, lower blue reflectance occurs on the shoreward, plume-influenced side of the boundary, where CDOM, organic particles, and phytoplankton associated with river discharge can all reduce blue reflectance [56,74,75]. An exception to that trend would be blooms of coccolithophorids, and recent evidence shows that dense surface concentrations of these organisms may lead to significant increases in optical backscattering and surface reflectivity [76]. The presence of coccolithophores has been microscopically confirmed as part of the phytoplankton species composition over the LTS [77], although generally at low abundance. Investigators have cautioned that suspended inorganic particles have spectral reflectivity patterns very similar to coccolithophore blooms [78,79]. These possibilities are not mutually exclusive; upwelling along shelf fronts may bring about the presence of both inorganic suspended particles and increased coccolithophore abundance.

In this particular case, apparent frontal boundary movement was detected by satellite where the prevailing zonal currents became perpendicular to the frontal boundary orientation (north/south). Over much of the LTS, the currents are instead aligned with the frontal boundary orientation and will not be as easily detected via gradients in ocean color image sequences. More advanced techniques that blend ocean color data with other data sources or utilize machine learning methods [80,81,82] may overcome this challenge. The optical front velocities estimated here from ABI (0.6–1.0 m s^−1^) were broadly consistent with the NCOM simulated velocities along the frontal boundary, although apparent optical front velocities and surface water current velocities are not the same quantities. Further work on satellite and model comparisons will require a much higher resolution physical model and high-frequency output retained for sub-hourly analysis.

It is the very high blue reflectance on the seaward side of the front that permitted a detection of contrast by GOES-ABI band 1. Nonetheless, the utility of ABI band 1 for this study was limited by the 1 km spatial resolution. The follow-on GOES sensors will have a finer spatial resolution for some visible bands [83], and the GLIMR mission will have a nadir resolution of 300 m [84]. This suggests that a combination of sensor datasets, including data blending methods, will permit oceanographic investigations at higher resolution scales of time and space. As new techniques emerge for blending ABI data (or other sensors) with ocean-color-calibrated sensors [85,86], there is a new opportunity to study and monitor ocean dynamics and the attendant biogeochemical consequences.

## 5. Conclusions

The high-temporal-resolution GOES-ABI data revealed the displacement of an optical boundary over the LTS that was interpreted, with NCOM support, as a frontal boundary. Coincident data provided by ocean-color-calibrated sensors confirmed that the identified boundary delineated two different optical regimes. The combination of sensors provided a more complete picture of the intraday frontal boundary movements: OLCI/VIIRS on polar-orbiting platforms for identification and confirmation of movement, and GOES-ABI providing five-minute imagery from which the multi-hour displacement was estimated. This study demonstrates that combining multiple sensors, including those not traditionally used for ocean observation, provides additional insight into smaller time/space-scale ocean processes.

Analysis of contemporaneous ocean model simulations provided a plausible physical explanation for the combined satellite observations. In the aftermath of a CAO event that consisted of a strong northerly wind, intense westward currents appeared along the frontal boundaries. NCOM displayed cyclonic relative vorticity associated with lateral shear on the seaward side of the modeled fronts, spatially adjacent to the satellite-observed high blue-band reflectance. This spectral reflectance contrast then identified a conspicuous westward movement of an apparent frontal boundary where the shelf widens and the simulated surface isopycnals diverge. The NCOM simulation is consistent with westward advection by the along-front current as a leading candidate explanation for the observed displacement. Daytime warming increased the modeled near-surface stratification and may have reduced frictional coupling, but its contribution to the frontal displacement was not isolated in this analysis. The combination of multiple-sensor data with ocean model analysis illustrates how candidate mechanisms for smaller-scale optical front displacement can be evaluated within the broader seasonal and regional context.

In this case, the strong contrast between the two optical regimes permitted feature tracking with ABI despite its limited signal-to-noise performance. Away from the coasts, lower-contrast offshore fronts may often be more difficult for ABI to detect. Future ocean color geostationary missions are designed to reduce this radiometric limitation [26]; however, temporal resolutions may still be restricted to hourly. This suggests that even with the advantage of ocean-color-calibrated and geostationary-based sensors, a combination of multiple sensors, including those not traditionally used or designed for ocean observation, may be useful tools to examine the cascading scales of time and space that cumulatively encompass a broad range of interrelated oceanographic phenomena.

## Data Availability

OLCI and SNPP VIIRS data are available from ESA, NOAA, and NASA at these locations: https://oceandata.sci.gsfc.nasa.gov and https://ladsweb.modaps.eosdis.nasa.gov, last accessed 15 December 2024. GOES/ABI data are available here: https://www.class.noaa.gov, last accessed January 2024.

## Supplementary Materials

The following supporting information can be downloaded at: https://www.mdpi.com/article/10.3390/s26175641/s1, Animation S1: GOES Color-enhanced LTS; Animation S2: GOES Color-enhanced front movement.
